# The Characteristics of Human Resources and Related Research Activities among Basic Stem Cell Research Groups in China

**Published:** 2019-01

**Authors:** Sheng-Jun WANG, Li-Juan LIU, Guang-Yue JI, Xiao-Hua (Andrew) ZHOU, Tian-Jiao JIANG, Mei-Hua LU, De-Lin YU, Qiang GUO, Jin-Hai SUN

**Affiliations:** 1. Department of Teaching, School of Medicine, Shanghai Children's Medical Center, Shanghai Jiaotong University, Shanghai, China; 2. Department of Health Management, Second Military Medical University, Shanghai, 200433, China; 3. Department of Logistics Support, Shanghai Tenth People’s Hospital, Shanghai, 200072, China; 4. Department of Training, Second Military Medical University, Shanghai, 200433, China; 5. Department of Biostatistics, School of Public Health, University of Washington, Washington, America; 6. Department of Medical Administration, Guilin Sanatorium of Guangzhou Military Command Area, Guilin, Guangxi, 541000, China

**Keywords:** Stem cell research, Human resources, Research activities, Influencing factors

## Abstract

**Background:**

This study aimed to evaluate the characteristics of faculty and research activities of basic stem cell research groups in China.

**Methods:**

A questionnaire was administered to persons who knew the information among 46 basic stem cell research groups in China. Multiple linear regression models and repeated-measures analyses of variance were used. Repeated-measures analyses of variance were used.

**Results:**

Of the 46 groups, 39.1% did not have any faculty recruited from abroad from 2009 to 2013, 37.0% did not have any faculty with junior-level title, 34.8% had ≤25.0% faculty with either M.D. or Ph.D. degree. Papers published in SCI journals per faculty and having faculty recruited from abroad were positively associated with research funding per faculty. The groups with faculty recruited from abroad had significantly higher research funding per faculty over time compared with the group without faculty recruited from abroad. Repeated-measures analyses of variance showed the group with faculty recruited from abroad had significantly higher research funding per faculty over time compared with the group without faculty recruited from abroad.

**Conclusion:**

To increase the development of basic stem cell research, some characteristics of human resources should be improved, and the groups should recruit more faculty with overseas experience.

## Introduction

Stem cell is one of the hottest areas in biomedical research (1). Although there are diverse ethical issues relevant to stem cell research, the field of stem cell research has continued to advance at a terrific pace over the past year (2). Stem cell research might deliver diverse medical benefits. By the research, we can learn more about why diseases develop and how they might be prevented or attacked (3). Some scientists claimed that stem cell research could generate cures and treatment for everything from heart disease to cancer (3).

Chinese government has provided abundant research funding to support related stem cell research (4). Because of policy support, many basic research groups engaged in stem cell research (5). China has made considerable progress in basic research of stem cells (4). Stem cell research in China is attracting more and more attention from around the world (1). Human resources and research funding were important for the progress of stem cell research in China. The team structure of 22 Chinese stem cell research groups were researched by interview, the organization of the research team would affect the scientific productivity (5).

However, few studies have focused on the human resources and research funding and scientific papers among stem cell research groups in China by quantitative research method. Our present study was carried out with three main objectives: to describe the number and characteristics of faculty in basic stem cell research groups in China, to examine research funding and scientific papers published in science citation index (SCI) journals from 2009 to 2013 in the groups, and to explore the factors predicting research funding and scientific papers published in SCI journals from 2009 to 2013 among the groups. We hope this research will allow readers to understand the current situation of human resources, funding and scientific papers published in SCI journals among basic stem cell research groups in China.

## Methods

The target subjects of this study were the basic stem cell research groups in China. We researched published papers on stem cell and searched the related institutional web site. There were about 98 groups engaged in basic stem cell research in China. All the groups were invited to participate in our research. Finally, 52 groups were willing to participate in the study. The 52 groups came from 13 institutes including Peking Union Medical College, Peking University, University of Chinese Academy of Sciences, Fudan University, Shanghai Jiaotong University, Tongji University, Second Military Medical University, Zhejiang University, and other universities.

A standardized questionnaire was sent to the related insiders of the 52 groups by e-mail, however, only 46 responded and completed the questionnaire thoroughly. The response rate of effective questionnaires was 88.5% (46/52).

The information on the group leader included the highest level of talent programs that he got (national talent programs, provincial and ministerial talent programs, other talent programs and no talent programs), whether the group leader was academician (yes, no), whether the group leader was Ministry of Science and Technology “973 Project Chief Scientist (yes, no), whether the group leader was the winner of National Science Foundation for Distinguished Young Scholars (yes, no), whether the group leader was the winner of National Science Foundation for Excellent Young Scholars (yes, no).

The information on faculty of the basic stem cell research groups included number of faculty, numbers of faculty with different highest degree (Doctor of Medicine (M.D.) or Doctor of Philosophy (Ph.D.), Master, Bachelor), numbers of faculty with different professional title (senior-level title, including professor or equivalent and associate professor or equivalent; middle-level title; junior-level title), whether had faculty recruited from abroad from 2009 to 2013 (yes, no). Other information included whether the group had scientific and technological collaboration with related hospitals (yes, no), whether had scientific and technological collaboration with companies (yes, no), whether had scientific and technological collaboration with institutes abroad (yes, no), the amount of research funding from 2009 to 2013, the number of research funding from 2009 to 2013, the number of papers published in SCI journals from 2009 to 2013.

### Data analysis

Descriptive statistical analysis was used to analyze the characteristics of the faculty of the 46 basic stem cell research groups in China. To determine the factors predicting the total amount of research funding from 2009 to 2013 and the total number of papers published in SCI journals from 2009 to 2013, multiple linear regression models with conditional stepwise analysis were used. Repeated-measures analyses of variance were used to identify changes in the amount of research funding over time between the groups with faculty recruited from abroad and the groups without faculty recruited from abroad. P<0.05 was considered significant. All data were analyzed with the SPSS 13.0 software (Chicago, IL, USA).

### Ethics approval

Informed consents were obtained from each participant. This study was approved by the Biological and Medical Ethics Committee, Second Military Medical University.

## Results

### The characteristics of group leaders

Academician, Ministry of Science and Technology “973 Project Chief Scientist, National Science Foundation for Distinguished Young Scholars, National Science Foundation for Excellent Young Scholars, national talent programs or provincial and ministerial talent programs winners were defined as excellent talents. Of the 46 group leaders, 38 (82.6%) were excellent talents.

### The characteristics of faculty

**Number of faculty.** Number of faculty ranged from 5 to 56 (mean = 16.57±1.45) for the 46 groups.

**Professional title of faculty.** Number of faculty with senior-level title ranged from 1 to 21 (mean = 2.87±0.53) for the 46 groups. Of the 46 project groups, 14 (30.4%) had ≤25.0% faculty with senior-level title, 21 (45.7%) had >25.0% and ≤50.0% faculty with senior-level title, 8 (17.4%) had >50.0% and ≤75.0% faculty with senior-level title, 1 (2.2%) had >75.0% and <100.0% faculty with senior-level title, 2 (4.3%) had 100.0% faculty with senior-level title. Number of faculty with middle-level title ranged from 0 to 10 (mean = 2.39±0.30) for the 46 groups. Of the 46 groups, 4 (8.7%) did not have any faculty with middle-level title. Number of faculty with junior-level title ranged from 0 to 10 (mean = 1.70±0.29) for the 46 groups. Of the 46 groups, 17 (37.0%) did not have any faculty with junior-level title (Table 1).

**Table 1:** Distributions regarding the professional title of faculty in the 46 groups

***Percentage of junior-level title faculty***	***Number of groups***	***Percentage***	***Percentage of middle-level title faculty***	***Number of groups***	***Percentage***	***Percentage of senior-level title faculty***	***Number of groups***	***Percentage***
0	17	37.0	0	4	8.7	0	0	0
>0 and ≤25.0	5	10.8	>0 and ≤25.0	9	19.6	>0 and ≤25.0	14	30.4
>25.0 and ≤50.0	19	41.4	>25.0 and ≤50.0	28	60.9	>25.0 and ≤50.0	21	45.7
>50.0 and ≤75.0	5	10.8	>50.0 and ≤75.0	5	10.8	>50.0 and ≤75.0	8	17.4
>75.0 and <100.0	0	0.0	>75.0 and <100.0	0	0.0	>75.0 and <100.0	1	2.2
100.0	0	0.0	100.0	0	0.0	100.0	2	4.3

**The highest degree of faculty.** The mean number of faculty with M.D. or Ph.D. degree was 6.07±0.69 for the 46 groups. Of the 46 project groups, 16 (34.8%) had ≤25.0% faculty with either M.D. or Ph.D. degree, 22 (47.8%) had >25.0% and ≤50.0% faculty with either M.D. or Ph.D. degree, 6 (13.0%) had >50.0% and ≤75.0% faculty with either M.D. or Ph.D. degree, 2 (4.3%) had 100.0% faculty with either M.D. or Ph.D. degree. The mean number of faculty with Master’s degree was 5.98±0.72 for the 46 groups. Of the 46 project groups, 2 (4.3%) did not have any faculty with Master’s degree, 12 (26.1%) had >0.0% and ≤25.0% faculty with Master’s degree, 24 (52.2%) had >25.0% and ≤50.0% faculty with Master’s degree, 8 (17.4%) had >50.0% and ≤75.0% faculty with Master’s degree. The mean number of faculty with Bachelor’s degree or less was 4.52±0.47 for the 46 groups. Of the 46 project groups, 4 (8.7%) did not have any faculty with Bachelor’s degree or less, 16 (34.8%) had >0.0% and ≤25.0% faculty with Bachelor’s degree or less, 23 (50.0%) had >25.0% and ≤50.0% faculty with Bachelor’s degree or less, and 3 (6.5%) had >50.0% and ≤75.0% faculty with Bachelor’s degree or less (Table 2).

**Table 2:** Distributions regarding the degree of faculty in the 46 groups

***Percentage of Bachelor’s degree***	***Number of groups***	***Percentage***	***Percentage of Master’s degree***	***Number of groups***	***Percentage***	***Percentage of Doctor’s degree or Ph.D***	***Number of groups***	***Percentage***
0	4	8.7	0	2	4.3	0	0	0
>0 and ≤25.0	16	34.8	>0 and ≤25.0	12	26.1	>0 and ≤25.0	16	34.8
>25.0 and ≤50.0	23	50.0	>25.0 and ≤50.0	24	52.2	>25.0 and ≤50.0	22	47.8
>50.0 and ≤75.0	3	6.5	>50.0 and ≤75.0	8	17.4	>50.0 and ≤75.0	6	13.0
>75.0 and <100.0	0	0.0	>75.0 and < 100.0	0	0	>75.0 and <100.0	0	0
100.0	0	0.0	100.0	0	0	100.0	2	4.3

Note: All the numerical value round to 1 decimal place. Therefore, the total percentage might not equal 100.0%.

**Faculty recruited from abroad.** Of the 46 groups, 18 (39.1%) did not have any faculty recruited from abroad from 2009 to 2013, 28 (60.9%) had faculty recruited from abroad.

### Scientific and technological collaboration

Of the 46 groups, 35 (76.1%) had scientific and technological collaboration with related hospitals, 30 (62.5%) had scientific and technological collaboration with related companies, and 44 (95.7%) had scientific and technological collaboration with institutes abroad.

### Research Funding and scientific papers published in SCI journals / Research Funding

The amount of research funding from 2009 to 2013 ranged from 0 to 42.69 million RMB (mean=11.21±1.34 million RMB) for the 46 groups. The number of funded projects from 2009 to 2013 ranged from 0 to 48 (mean=9.59±1.25) for the 46 groups. Distributions regarding the number of funded projects from 2009 to 2013 were examined to be 2 (4.4%) for 0 funded projects, 15(32.6%) for 1–5 funded projects, 10 (21.7%) for 6–10 funded projects, 19 (41.3%) for ≥11 funded projects.

In Table 3, factors predicting the amount of research funding from 2009 to 2013 per faculty among the 46 groups were shown. High number of papers published in SCI journals from 2009 to 2013 per faculty and having faculty recruited from abroad were positively associated with the amount of research funding from 2009 to 2013 per faculty. Descriptive statistics between the group with faculty recruited from abroad and the group without faculty recruited from abroad were listed in Table 4 and were based on the amount of research funding per faculty at year 2009 and at the subsequent 4 yr. The group with faculty recruited from abroad had significantly higher research funding per faculty over time compared with the group without faculty recruited from abroad (*P*=.017).

**Table 3:** The factors predicting the research funding from 2009 to 2013 per faculty among the 46 groups. Results of multivariate, stepwise linear analysis

***Factor***	***B***	***Beta***	***P value***
Constant	−30.959	-	.285
The mean number of papers published in SCI journals from 2009 to 2013 per faculty	59.136	.416	.002
Whether officially recruited faculty from abroad from 2009 to 2013 ^a^	43.334	.348	.009
Whether had excellent talent leaders ^b^	-	−.071	.608
The percentage of faculty with either M.D. or Ph.D. degree	-	−.024	.864
The percentage of faculty with senior-level title	-	.052	.714
Whether had scientific and technological collaboration with related clinical units ^c^	-	.003	.982
Whether had scientific and technological collaboration with companies ^d^	-	−.108	.439
Whether had scientific and technological collaboration with institutes abroad ^e^	-	−.139	.291

NOTE: B = Unstandardized Coefficients; Beta = Standardized Coefficients. //

a No = 1; yes = 2.

b No = 1; yes = 2. //

c No = 1; yes = 2.

d No = 1; yes = 2. //

e No = 1; yes = 2.

**Table 4:** Mean research funding per faculty at 5-year point assessments

***Year***	***With faculty officially recruited from abroad (mean±SE)***	***Without faculty officially recruited from abroad (mean±SE)***	***P^b^***
2009	9.61±2.40	3.72±1.24	.017
2010	13.56±3.46	8.79±4.08	
2011	23.81±7.16	10.91±4.60	
2012	23.88±5.95	7.09±1.78	
2013	18.23±2.97	14.91±2.70	

Note:

a The means research funding per faculty at all time points from 2009 to 2013 were compared in repeated-measures analyses of variance to test the interactions between time and official recruitment of faculty from abroad.

**Fig. 1:**
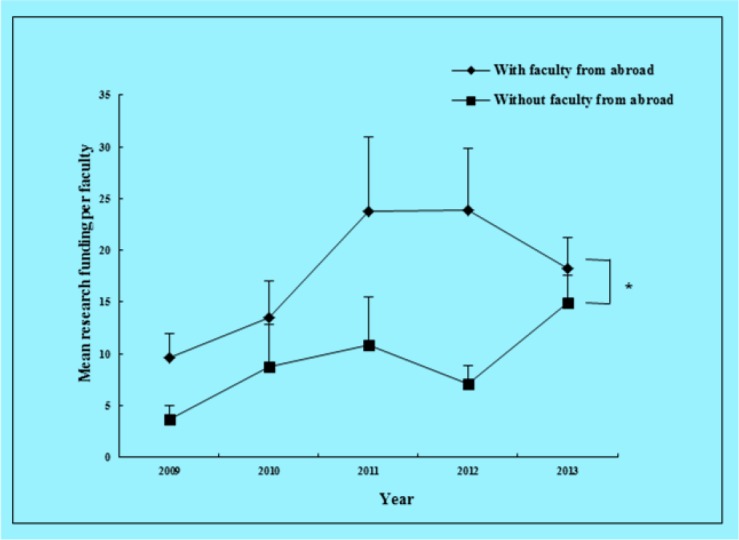
Mean research funding per faculty at 5-year Point Assessments Significant differences were observed between the 2 groups over the period (*P* =.017)

Figures were generated to illustrate visually and clearly the changing trend in mean research funding per faculty from 2009 to 2013 between the group with faculty recruited from abroad and the group without faculty recruited from abroad (Fig. 1). There was difference in the amount of research funding per faculty from 2009 to 2013 between the group with faculty recruited from abroad and the group without faculty recruited from abroad.

### The number of scientific papers published in SCI journals

The total number of scientific papers published in SCI journals from 2009 to 2013 ranged from 0 to 45 (mean=10.15±1.47) among the 46 groups. Of the 46 groups, 5 (10.9%) had not published any scientific papers in SCI journals from 2009 to 2013, 15 (32.6%) had published 1–5 scientific papers in SCI journals, 8 (17.4%) had published 6–10 scientific papers in SCI journals, 7 (15.2%) had published 11–15 scientific papers in SCI journals, 6 (13.0%) had published 16–20 scientific papers in SCI journals, 5 (10.9%) had published ≥21 scientific papers in SCI journals.

The factors predicting the number of scientific papers published in SCI journals from 2009 to 2013 per faculty among the 46 groups are shown in Table 5. The number of research funding from 2009 to 2013 per faculty were positively associated with the number of scientific papers published in SCI journals from 2009 to 2013 per faculty.

**Table 5:** The factors predicting the number of papers published in SCI journals from 2009 to 2013 per faculty among the 46 groups. Results of multivariate, stepwise linear analysis

***Factor***	***B***	***Beta***	***P-value***
Constant	.350	-	<.001
The research funding from 2009 to 2013 per faculty	.003	.418	.004
Whether had excellent talent leaders ^a^		.067	.631
The percentage of faculty with either M.D. or Ph.D. degree		−.122	.388
The percentage of faculty with senior-level title		.152	.271
Whether officially recruited faculty from abroad from 2009 to 2013 ^b^		−.160	.279
Whether had scientific and technological collaboration with related clinical units ^c^		.033	.812
Whether had scientific and technological collaboration with companies ^d^		.100	.473
Whether had scientific and technological collaboration with institutes abroad ^e^	-	.193	.162
The 5-year number of research funding per faculty		.181	.336

NOTE: B = Unstandardized Coefficients; Beta = Standardized Coefficients.

a No = 1; yes = 2.

b No = 1; yes = 2.

c No = 1; yes = 2.

d No = 1; yes = 2.

e No = 1; yes = 2.

## Discussion

Stem cell research remains a tremendously promising field of study (6). It continues to attract considerable public interest (6). Leaders in research and development organizations performed important roles within project groups that contributed significantly to performance (7). A few leadership roles were essential for innovation (8) including idea generating, entrepreneuring/championing, project leading, gatekeeping, and sponsoring/coaching (9). Overall, the ability of the group leaders was very important. Our research suggested that most of the leaders in stem cell research groups were excellent talents. It would contribute significantly to the performance of stem cell research in China.

The characteristics of faculty were also important for the development of basic stem cells research groups, such as the distributions of professional title and highest degree. Our study showed that 37.0% of the 46 groups did not have any faculty with junior-level title, 8.7% did not have any faculty with middle-level title, and 4.3% had 100.0% faculty with senior-level title. The structure of professional titles was not ideal for some of the groups engaged in basic stem cell research. The structure of professional titles should be improved for some groups.

About the highest degree of faculty, our research showed that 6.5% of the 46 groups had >50.0% and ≤75.0% faculty with Bachelor’s degree or less, and 34.8% had ≤25.0% faculty with either M.D. or Ph.D. degree. Overall, 83% of the faculty had M.D. degrees, 8% had a Ph.D. degree, and 7% have both degrees (10). The education level of the faculty in some basic stem cell research groups should be improved in China.

The collaboration between basic research groups and hospitals and the collaboration between basic research groups and companies could accelerate the transformation from basic research to application. Our results indicated most of the basic stem cell research groups had scientific and technological collaboration with related hospitals and companies, which was helpful to the transformation from basic stem cell research to application. Science and engineering research has become an increasingly international phenomenon. A study analyzed over 2,800 articles from the top journals that include stem cell research in their publications showed that international collaborations increased from 20.9% to 36% from 2000 to 2010 (11). Our research showed 95.7% of the basic stem cell research groups had scientific and technological collaboration with institutes abroad, which demonstrated the globalization of stem cell science in China.

Funding had been acknowledged as one of the main drivers of scientific activities (12). Research funding was important for the development of stem cell research groups. Most of the basic stem cell research groups had projects supported by research funding from 2009 to 2013, only 4.4% of the groups did not have any projects supported by Research Funding. To examine the factors influencing the amount of research funding among the basic stem cell research groups, a multiple linear regression model with conditional stepwise analysis was used. The number of papers published in SCI journals from 2009 to 2013 per faculty and having faculty recruited from abroad was positively associated with the amount of research funding from 2009 to 2013 per faculty. Funding agencies wanted to see their limited resources have a bigger impact and researchers need increased productivity to compete for highly-prized research grants (12). A research measured some factors associated with the amount of funding, which indicated that past productivity of researchers positively affected the funding level (13). Studies with lower chances of publishing in high visibility journals were not deemed worthy of obtaining a grant from formal sources or that the grant offer was severely limited (14). It was consistent with our finding. The possible explanation might be that if the faculty had more related past productivity, for example, publications, he would be considered to have more research capacity. Therefore, the faculty with more past productivity would get more funding.

It was common for health and health-related professionals to study abroad. The impact of overseas experience on research capacity of Chinese health professionals indicated that the number of National Natural Science Foundation of China among the group with overseas experience was significantly higher than that among the group without overseas experience (15). It was consistent with the results of our study. The results indicated the benefits of recruiting faculty from abroad for the basic stem cell research groups. Of the 46 groups, more than one third did not have any faculty recruited from abroad from 2009 to 2013. Basic stem cell research groups should be encouraged to recruit more faculty with overseas experience, especially for those groups without any faculty recruited from abroad.

## Conclusion

The number of papers published in SCI journals from 2009 to 2013 per faculty and having faculty recruited from abroad were positively associated with the amount of research funding. To increase the development of basic stem cell research, they should be encouraged to recruit more faculty with overseas experience, especially for those groups without any faculty recruited from abroad.

## Ethical considerations

Ethical issues (Including plagiarism, informed consent, misconduct, data fabrication and/or falsification, double publication and/or submission, redundancy, etc.) have been completely observed by the authors.
